# BUILDING OUR LARGEST DEMENTIA (BOLD) INFRASTRUCTURE FOR ALZHEIMER'S ACT: CENTERS OF EXCELLENCE

**DOI:** 10.1093/geroni/igac059.274

**Published:** 2022-12-20

**Authors:** Lisa McGuire

**Affiliations:** CDC, Atlanta, Georgia, United States

## Abstract

While public health experts cannot yet say how to prevent Alzheimer’s disease and related dementias (ADRD), emerging science indicates that ADRD may be slowed through the implementation of risk reduction strategies, early diagnosis, and better education and training of front-line health care professionals. The Building Our Largest Dementia (BOLD) Infrastructure for Alzheimer's Act was introduced into the Senate (S. 2076) on November 6, 2017 and signed into law on December 31, 2018 (P.L. 115-406) amending the Public Health Service Act (Section 398A; 42 U.S.C. 280c-3-4). CDC's Alzheimer's Disease and Healthy Aging Program (AD+HAP) was authorized to implement the activities outlined in BOLD which were designed to create a uniform national public health infrastructure. This presentation will provide an overview of BOLD and the demonstrate how BOLD Program and Public Health Centers of Excellence recipients are working together to build a uniform national public health infrastructure.

